# Evaluation of Selenoprotein Supplementation on Digestibility, Growth, and Health Performance of Pacific White Shrimp *Litopenaeus vannamei*

**DOI:** 10.1155/2023/2008517

**Published:** 2023-01-05

**Authors:** Rafi Kemal, Ichsan Achmad Fauzi, Sri Nuryati, Wira Wisnu Wardani, Muhammad Agus Suprayudi

**Affiliations:** ^1^Department of Aquaculture, Faculty of Fisheries and Marine Sciences, IPB University, Bogor 16680, Indonesia; ^2^PT Aquacell Indo Pasifik, Jl. Pedurenan 5, Gunung Sindur, Bogor 16340, Indonesia

## Abstract

Selenoprotein is a feed additive that can overcome oxidative stress in intensive Pacific white shrimp (*Litopenaeus vannamei*) culture. This study evaluated the effects of selenoprotein supplementation at various doses on Pacific white shrimp's digestibility, growth, and health performance. The experimental design used was a completely randomized design consisting of four feed treatments, namely, control and treatments with selenoprotein supplementation of 2.5, 5, and 7.5 g kg feed^−1^ with four replications. Shrimps (1.5 g) were reared for 70 days and challenged for 14 days by the bacteria *Vibrio parahaemolyticus* (10^7^ CFU mL^−1^). Shrimps used in the digestibility performance evaluation (6.1 g) were reared until sufficient quantities of feces were collected for analysis. Shrimp supplemented with selenoprotein exhibited superior digestibility, growth, and health performance compared to the control (*P* < 0.05). The use of selenoprotein at a dose of 7.5 g kg of feed^−1^ (2.72 mg Se kg of feed^−1^) was considered the most effective for increasing productivity and preventing disease attacks in intensive shrimp culture.

## 1. Introduction

The Pacific white shrimp or whiteleg shrimp *Litopenaeus vannamei* is the most valuable aquaculture commodity globally. According to FAO [1] data, the production value of whiteleg shrimp in 2018 reached 30,222 million USD accounting for 11.5% of the total world aquaculture production value, with total production reaching 4.97 million tons (4.3% of the total world aquaculture production). The high production cannot be separated from the uniqueness of whiteleg shrimp, which has increased market value, high resistance to diseases, rapid growth rate, and adaptation to diverse culture systems [2]. Whiteleg shrimp production depends on the use of feed with the right content for shrimp needs to optimize growth [3].

The whiteleg shrimp farmers apply intensive aquaculture systems to optimize production to meet the ever-increasing global market demand. The application of this intensive aquaculture system may be detrimental to the aquaculture environment due to the accumulation of organic waste and the metabolism of whiteleg shrimp [4]. The high concentration of aquaculture waste can cause oxidative stress in whiteleg shrimp by increasing the concentration of reactive oxygen species (ROS) [5]. ROS, including superoxide anions (O_2_^−^), hydroxyl radicals (OH^−^), and hydrogen peroxide (H_2_O_2_), are by-products of aerobic metabolism [6] produced during disease challenge and oxidative deamination [7, 8]. Superoxide anion is the first by-product of oxidative stress and will be produced continuously by aerobic metabolism; it can damage vital biological molecules in the body [9]. Oxidative stress can reduce the body's defense system and shrimp's appetite, inhibiting molting and growth and reducing resistance to *Vibrio* bacteria and mass mortality [10–12]. The combination of intensive shrimp farming systems (high density), poor quality of the culture environment, and infection with pathogens will result in outbreaks of bacterial diseases, such as vibriosis and AHPND (Acute Hepatopancreatic Necrosis Disease) caused by the toxin strain of *Vibrio parahaemolyticus* [13, 14]. AHPND-infected shrimp exhibit lethargy, appetite loss, an empty stomach and middle intestine, pale-to-white hepatopancreas atrophy, and mass mortality rate that can exceed 70% [15, 16]. These conditions can disrupt production and result in economic losses for whiteleg shrimp farms.

Farmers can supplement feed additives to feed to address oxidative stress in intensive shrimp culture systems. One type of feed additive that can be used is selenoprotein. Selenoprotein is composed of both selenium (Se) and peptides. Selenium is an essential trace element for growth and physiological functions in cultured fish as well as crustaceans and is an essential component of feed [17, 18]. Selenium plays a role in the glutathione peroxidase enzyme to reduce and destroy peroxides that damage body cells and tissue synergy to form antioxidants that can boost the immune system [19]. Selenium also plays a role in the iodothyronine deiodinase enzyme's ability to increase the production of the hormone thyroxine, which affects the increase in IGF (insulin-like growth factor) [20, 21]. This increase in IGF will lead to a rise in protein and carbohydrate metabolism, which has implications for increasing nutrient absorption, feed intake, and fish growth [17].

Selenoprotein is one of the most potent forms of selenium that can be used as a shrimp selenium supplement. It readily binds to the peptides (proteins) that compose the body. Organic selenium is better absorbed by the body, easier to digest, more biologically active, and more tolerable than inorganic selenium [17, 22]. Selenoproteins contain peptides that are composed of two or more amino acids. According to [23], whiteleg shrimp absorb amino acids in the form of peptides more efficiently than they absorb single amino acids. Moreover, it was reported that the physical and chemical properties of peptides were superior to those of single amino acids. This study was designed to evaluate the selenoprotein supplementation with various doses on the digestibility, growth, and health performance of whiteleg shrimp.

## 2. Materials and Methods

### 2.1. Diet Preparation and Dietary Treatments

This experiment consisted of three stages: an evaluation of digestibility, growth, and health performance before and after the *Vibrio parahaemolyticus* challenge test. Digestibility performance analysis was carried out separately from other parameter tests. The experimental design used was completely randomized. The analysis of digestibility and growth performance was conducted with four feed treatments and four replications, including the control treatment (without selenoprotein supplementation) and selenoprotein supplementation at doses of 2.5, 5, and 7.5 g kg of feed^−1^. The challenge test consisted of five feeding treatments and three replications. The four treatments listed above were tested against *V*. *parahaemolyticus* bacteria and the negative control treatment by shrimp (without a challenge test). The test feed utilized in this study was commercial feed with a dry protein and fat content of 35% and 6.06%, respectively, coated with selenoprotein. This research was using commercial feed as a basal diet to replicate common supplementation practices that have been used by shrimp farmers. The selenoprotein is a product developed by PT. Aquacell Indo Pasifik marketed under the brand name AQUACELL GF Shrimp®. The procedure for making treatment feed is based on the protocol of the AQUACELL GF Shrimp® product. Selenoprotein was weighed and diluted with water as much as 160 mL kg of feed^−1^. Then, the selenoprotein solution was added to the feed and stirred until the feed mixture is evenly distributed. To make sure that selenoprotein were evenly distributed in the feed, the feed mixture was then stored at airtight conduction for 24 hours in accordance with the manufacturer's protocol.

There is an additional procedure for preparing test feed for shrimp digestibility performance when selenoprotein has not been supplemented to the feed. Commercial feed was ground by a grinder with mesh size of 2.38 mm. The grinded feed was then mixed with a binder in the form of polymethyl carbamide (PMC) and Cr_2_O_3_ as digestibility marker. The resulting mixture contained 99.1% commercial feed, 0.6% Cr_2_O_3_, and 0.3% PMC. The feed was then shaped into pellets with a pellet machine (1.0 mm die hole) and dried in an oven at 50°C for 4 hours. The feed was supplemented with selenoprotein in accordance with the trial. For the feeding and disease trials, the commercial feed was directly mixed with different dosages of selenoprotein without any grinding and pelleting. The feed and selenoprotein mixtures were then stored in an airtight container for 24 hours before being fed to the shrimp. The proximate analysis of feed included measurement of moisture, protein, fat, ash, crude fiber, and nitrogen-free extract using the AOAC [24] method in the laboratory of Fish Nutrition, Department of Aquaculture, IPB University. The analysis of chromium oxide (Cr_2_O_3_) used spectrophotometry as performed by Mcginnis and Kasting [25]. Selenium analysis of the test feed was carried out using ICP-OES (Inductively Coupled Plasma–Optical Emission Spectrometry) in Saraswanti Indo Genetech Bogor's laboratory, Indonesia. The results of the analysis of proximate and selenium in test feed for the growth performance of selenoprotein-supplemented whiteleg shrimp are presented in Table 1.

### 2.2. Shrimp and Experimental Set-Up

Specific Pathogen Free (SPF) pacific white shrimp were obtained from the Carita Hatchery unit of PT. Suri Tani Pemuka Anyer, Banten Province, Indonesia. Post larvae 8 of whiteleg shrimp fry were reared to the required size for the study in a 6 × 3 × 1.5 m concrete container with a 1 m water depth (volume 18 m^3^) at IPB Fisheries and Marine Observation Station (IFMOS) Ancol, North Jakarta. This experiment consisted of three stages: feces collection (digestibility performance), 70-day culture (growth performance), and challenge test (health performance).

The whiteleg shrimp used for digestibility performance analysis had a body weight of 6.1 g individual^−1^ and density of 15 shrimp per tank. The tanks used were 16 units measuring 90 × 40 × 45 cm with a 22 cm water depth (volume 79.2 L). The tank is equipped with an aeration system, thermostat, and physical filter and is kept in a UV plastic sealed room. The shrimp were fed twice daily (07.00 and 15.00) at satiation. For digestibility analysis, the feces were collected by siphoning using a small tube and then filtered through a fabric filter until sufficient quantities were obtained for analysis. The feces were placed in a tightly sealed container and frozen at -20°C. The feces are oven-dried for four to six hours at 50°C. Furthermore, the Cr_2_O_3_ and proximate analysis of the feces were conducted using the Mcginnis and Kasting [25] method and AOAC [24] methods.

The whiteleg shrimp used for growth performance analysis had an average body weight of 1.5 g individual^−1^ and density of 15 shrimp per tank. The tank used mirrored those of the shrimp digestibility performance analysis. Whiteleg shrimp were given test feed five times per day (06.00, 10.00, 14.00, 18.00, and 22.00) for 70 days of rearing with an initial feeding rate of 8% of the shrimp biomass. After rearing with treated feed, ten shrimp per tank were intramuscularly injected with *Vibrio parahaemolyticus* at a density of 10^7^ CFU mL^−1^ with a volume of 100 *μ*L shrimp^−1^. The negative control (NC) shrimp were injected with a PBS solution. Shrimps were then reared for 14 days on treatment feed, and the number of shrimps that died was determined as shrimp survival data at the end of the challenge test.

Water quality management is done by changing water to ensure optimum water quality values for shrimp growth. Every four days, 30 percent of the water exchange was conducted by siphoning the feces and uneaten feed at the bottom of the tank, and then, the new water was added into the tank by water hose. During the experiment, the total water volume in each tank was maintained uniformly, and aeration was run continuously. During the study, water quality parameters were measured: temperature of 28.0–30.0°C, pH 7.0–7.5, salinity 23–25 g/L, DO ≥ 4.0 mg/L, TAN < 0.1 mg/L, NO2 − <0.1 mg/L, and alkalinity ≥ 120 mg/L.

### 2.3. Shrimp Sample Collection

Before rearing shrimp for growth performance analysis, 20 shrimp were randomly selected for proximate analysis of the initial shrimp body. After 70 days of feeding with the treated feed, the shrimp were fasted for 24 hours. Then, the final weight was determined, and three shrimps (per tank) were sampled for proximate body analysis. A total of 3 × 3 shrimp per treatment (three replicates) had hemolymph extracted from the base of the swimming legs using a one mL syringe to create two 0.8 mL tubes (0.4 mL hemolymph and 0.4 mL anticoagulant). The anticoagulant used was Inviclot® Heparin Sodium 5000 IU. The shrimp's hemolymph was used to test its immune response. The shrimp were surgically dissected, and the hepatopancreas was removed to determine the antioxidant activity. Anesthesia (when removing hemolymph and injecting pathogenic bacteria into the shrimp body) used was Clove Bud Oil from PT. Tamba Sanjiwani at a dose of 200 *μ*L L^−1^ seawater using immersion method. The same quantity of hemolymph and the hepatopancreas was collected from each treatment on the second day (48 hours after bacterial injection) postchallenge test. The initial and final shrimp samples were stored in a -20°C freezer, while hemolymph and the hepatopancreas samples were kept in a -80°C freezer.

### 2.4. Observation Parameters

#### 2.4.1. Apparent Digestibility

Similar to the growth and health experiment, apparent digestibility coefficients (ADC) of dry matter, protein, and energy were determined using the different shrimp in a bioassay for each experimental diet. The ADCs of dry matter (DM), protein, and energy were calculated as follows for each experimental diet:

- ADC of dry matter (%) = [1 − (%Cr_2_O_3_ in feed/%Cr_2_O_3_ in feces)] × 100
- ADC of protein&energy (%) = [1 − ((%*Cr*_2_*O*_3_ in *feed*/%*Cr*_2_*O*_3_ in feces) × (%protein or energy in feces/%protein or energy in feed) )] × 100

#### 2.4.2. Growth Performance

After the feeding trial, shrimp were collected, counted, and group-weighed per aquarium. The parameters for shrimp growth, nutrient utilization, survival, and economic conversion value were calculated as follows:

- The growth parameters such as specific growth rate (SGR; %g day^−1^) = 100 × [*t*√(*W*_2_/*W*_1_) − 1] and average daily growth (ADG; g day^−1^) = [*W*_2_ − *W*_1_]/*t*, where *W*_2_ is the final weight, *W*_1_ is the initial weight, and *t* is the experimental period
- Nutrient utilization parameters such as feed intake = the sum of the feed offered to shrimp during the experiment; dry feed intake = feed intake − (feed intake × %feed moisture); feed conversion ratio (FCR) = feed intake/biomass gain; dry FCR = dry feed intake/biomass gain; protein efficiency ratio (PER) = biomass gain/protein intake; and protein retention (PR) = body protein content gain/protein intake
- Survival rate (SR; %) = 100 × (*N*_*t*_/*N*_0_), where *N*_*t*_ is the number of live shrimps at the end and *N*_0_ is the number of live shrimps at the beginning of the study
- Economic conversion ratio (ECR; %) = price of treatment feed per kg × dry FCR

#### 2.4.3. Immune Response, Antioxidant Activity, and Resistance of Shrimp

Among the immune response test parameters observed are the total hemocyte count (THC), phagocytic activity (PA), respiratory burst (RB), phenoloxidase activity (PO), lysozyme activity (LA), and immunity-related gene expression. In addition, the antioxidant activity, which includes superoxide dismutase (SOD), malondialdehyde (MDA), and glutathione peroxidase (GPx), was measured after 70 days of rearing (prior to the challenge test) and on the second-day postchallenge test (48 hours after bacterial injection), as well as the survival rate (SR) (14 days after challenge test).

*(1) Total Hemocyte Count (THC)*. THC was calculated to discover the number of the shrimp hemocytes based on Wang and Chen's [27] method. THC was observed for the number of cells on a hemocytometer using a microscope and calculated as follows:

(1) $$\begin{array}{c} \text{THC}=\frac{\sum \text{o}\text{bserved cell}}{\sum \text{o}\text{bserved box}}\times 25\times \frac{1}{\text{volume of hemacytometer}}\times \text{dilution factor}. \end{array}$$

*(2) Phagocytic Activity (PA)*. Phagocytic activity was determined using the method of Anderson and Siwicki [28] with *Staphylococcus aureus* 10^7^ CFU mL^−1^ as the test bacteria. Phagocytic activity was calculated based on the percentage of 100 phagocytic cells exhibiting phagocytic activity using the following formula:

(2) $$\begin{array}{c} \text{Phagocytic activity }\left(\%\right)=\frac{\text{the number of cells that carry out phagocytic }}{\text{the number of phagocytic cells}}\times 100. \end{array}$$

*(3) Respiratory Burst (RB)*. The respiratory burst activity was evaluated using the reduction of nitro blue tetrazolium (NBT) per 10 *μ*L hemolymph as a measurement of superoxide anions (O_2_^−^) based on the study of Song and Hsieh [29]. The RB activity was measured using a microplate reader/spectrophotometer at a wavelength of 630 nm.

*(4) Phenoloxidase (PO)*. The phenoloxidase activity was measured using the method of Liu and Chen [30] based on the formation of dopachrome produced by L-dihydroxy phenylalanine (L-DOPA). The PO activity was measured using a microplate reader/spectrophotometer at a wavelength of 490 nm.

*(5) Lysozyme Activity (LA)*. The lysozyme activity was measured using test bacteria *Micrococcus lysodeikticus* according to the [31]) method. The LA activity was measured using a microplate reader/spectrophotometer at a wavelength of 450 nm for 0 and 30 minutes of mixing.

*(6) Immune Gene Expression*. The expression of the immune-related genes was analyzed using quantitative real-time reverse transcription- (RT-) polymerase chain reaction (PCR). The total RNA of the hemocytes was extracted and purified using GENEzol™ reagent (Geneaid, Taiwan). The first-strand complementary DNA (cDNA) was synthesized from total RNA using ReverTra Ace® qPCR RT Master Mix with gDNA Remover (Toyobo Co., Ltd., Japan). The following specific primer pairs were designed to quantify the shrimp immune genes of prophenoloxidase (proPO), peroxinectin (PE), and housekeeping gene *β*-actin: proPO forward primer 5′-GCC-TTG-GCA-ACG-CTT-TCA-3′ and reverse primer 5′-CGC-GCA-TCA-GTT-CAG-TTT-GT-3′ [32]; PE forward primer 5′-TGG-ACC-TCG-CGG-GAG-AT-3′ and reverse primer 5′-GAC-CGA-TAG-CCA-CCA-TGC-TT-3′ [33]; and *β*-actin forward primer 5′-GAG-CAA-CAC-GGA-GTT-CGT-TGT-3′ and reverse primer 5′-CAT-CAC-CAA-CTG-GGA-CGA-CAT-GGA-3′ (GenBank accession no.: AF300705).

The RT-PCR analysis was conducted using KAPA SYBR® FAST qPCR (Kapa Biosystems, Inc., USA) on a Rotor-Gene 6000 machine (Corbett, USA). Amplification was performed in a 20 *μ*L reaction volume containing ten *μ*L of the FAST SYBR*®* qPCR Green Master Mix, 1.6 *μ*L (0.8 *μ*L each of the forward and reverse primers), four *μ*L of cDNA template, and 4.4 *μ*L of nuclease-free water (NFW). Untreated material (NC) was NFW. The qPCR program used was predenaturation at 95°C for 2 minutes, followed by 40 cycles at 95°C for 10 seconds (denaturation), 60°C for 15 seconds (annealing) and performed acquiring to cycling A, 72°C for 15 seconds (extension), and melting curve at range temperature 72–95°C. The RT-PCR data analysis was carried out using Rotor-Gene Q software (Rotor-Gene QIAGEN) in accordance with the [14]) method.

*(7) Superoxide Dismutase (SOD), Malondialdehyde (MDA), and Glutathione Peroxidase (GPx)*. Analyses of SOD, MDA, and GPx were performed on 0.15 g of shrimp hepatopancreas samples. SOD analysis was carried out using SOD colorimetric test kit brand Abcam, UK, according to McCord and Fridovich [34]. The standard used in this analysis is the enzyme SOD (EC 1.15.1.1). MDA analysis was carried out using MDA lipid peroxidation colorimetric test kit brand Abcam, UK, as stated by [35]. GPx analysis was carried out by employing a glutathione peroxidase activity colorimetric assay kit brand Abcam, UK.

#### 2.4.4. Proximate Analysis

Proximate analysis was carried out on feed samples and the shrimp bodies at the beginning and the end of rearing based on the method of AOAC [24]. Analysis of moisture content was done by oven-heating method at 105–110°C for 12 hours. Protein analysis was done by Kjeldahl method. Crude fiber analysis was done by dissolving weak acids, weak bases, and organic solvents. Analysis of ash content was done by heating the furnace to 600°C. Fat analysis of feed was done using Soxhlet's method and body fat using Folch's method [26].

### 2.5. Data Analysis

The research data were processed and analyzed using Microsoft Excel 2016 and SPSS version 16.0. The data were examined for normality and homogeneity prior to the analysis of variance. Analysis of variance (ANOVA) at a 95% confidence interval was used to determine the significant difference between the test parameter treatments. If the results are significantly different, Duncan's further test is used to determine the significant difference between treatments.

## 3. Results

### 3.1. Apparent Digestibility

The ADC of whiteleg shrimp fed with various doses of selenoprotein is presented in Table 2. Based on the results of two-way ANOVA, the digestibility performance of whiteleg shrimp supplemented with selenoprotein was significantly different (*P* < 0.05) from that of the control. ADC of dry matter of whiteleg shrimp fed with selenoprotein supplementation at doses of 5 and 7.5 g kg of feed^−1^ showed significantly higher *P* < 0.05 than the control, while the treatment at a dose of 2.5 g kg of feed^−1^ did not differ significantly (*P* > 0.05) from the control. The ADC of protein of whiteleg shrimp supplemented with selenoprotein at a dose of 7.5 g kg of feed^−1^ was significantly higher (*P* < 0.05) than the control, whereas the other doses were not significantly different (*P* > 0 0.05) from the control. The ADC of energy of whiteleg shrimp fed with selenoprotein supplementation showed a significantly higher *P* < 0.05 than the control.

### 3.2. Growth Performance

The growth performance of whiteleg shrimp fed with various doses of selenoprotein for seventy days is shown in Table 3. Based on the results of two-way ANOVA, the growth performance of whiteleg shrimp supplemented with selenoprotein was significantly different (*P* < 0.05) from that of the control. Final biomass, final body weight, SGR, and ADG of whiteleg shrimp fed with selenoprotein supplementation at a dose of 7.5 g kg of feed^−1^ showed significantly higher *P* < 0.05 than the control, while the other doses were not significantly different (*P* > 0.05) from the control. The feed intake, PER, and PR of whiteleg shrimp fed with selenoprotein supplementation exhibited significantly higher *P* < 0.05 than the control as the selenoprotein dose in the feed increased. FCR and dry FCR of whiteleg shrimp fed with selenoprotein supplementation at a dose of 7.5 g kg of feed^−1^ was significantly better (*P* < 0.05) when compared to other treatments. Dry feed intake in all treatments did not differ significantly (*P* > 0.05). SR of whiteleg shrimp fed with selenoprotein supplementation at a dose of 7.5 g kg of feed^−1^ was superior to that of other treatments.

The economic conversion ratio was demonstrated in Table 4. Supplementation of 2.5, 5, and 7.5 g kg feed^−1^ of selenoprotein will cost 0.020, 0.041, and 0.061 USD. However, in the case of ECR, the lowest ECR were found in the 7.5 g kg feed^−1^. It was demonstrated also in the ECR parameter that all the treatment with selenoprotein supplementation produce lower feed cost compared to the control.

### 3.3. Immune Response, Antioxidant Activity, and Resistance of Shrimp

The health performance of whiteleg shrimp fed with selenoprotein treatment at varying doses for 70 days and challenged with *V*. *parahaemolyticus* for 14 days consisted of three tests: immune response, antioxidant activity, and resistance of shrimp. The whiteleg shrimp's immune response is presented in Table 5. Based on the results of two-way ANOVA, the immune response of whiteleg shrimp supplemented with selenoprotein supplementation was significantly better than the control (*P* < 0.05), both prior to and postinfection with *V. parahaemolyticus* bacteria. The whiteleg shrimp fed with selenoprotein supplementation had significantly higher values of THC, PO, RB, and LA (*P* < 0.05) compared to the controls as the dose of selenoprotein in the feed increased, and the PA value of whiteleg shrimp treated with selenoprotein was significantly higher (*P* < 0.05) than that of the control group. In all treatments, the THC, PO, RB, PA, and LA values of whiteleg shrimp decreased 48 hours postchallenge test. The values of THC, RB, PA, and LA in the postchallenge test of whiteleg shrimp fed with selenoprotein supplementation were significantly (*P* < 0.05) higher than the positive control (PC) as the dose of selenoprotein in the feed increased. The PO value of whiteleg shrimp postchallenge test fed with selenoprotein supplementation at doses of 5 and 7.5 g kg of feed^−1^ was significantly higher (*P* < 0.05) than the PC, while the selenoprotein treatment at a dose of 2.5 g kg of feed^−1^ did not differ significantly (*P* > 0.05) from the PC. Survival rate (SR) of whiteleg shrimp exposed to *V*. *parahaemolyticus* bacteria and supplemented with selenoprotein was significantly higher (*P* < 0.05) than PC.

The relative expression value of mRNA immune genes of whiteleg shrimp is presented in Table 5. Based on the results of two-way ANOVA, the relative expression values of the proPO and PE genes of whiteleg shrimp postchallenge fed with selenoprotein supplementation at a dose of 7.5 g kg of feed^−1^ were significantly higher (*P* < 0.05) than the PC and other doses. The antioxidant activity of whiteleg shrimp is shown in Table 5. Prior to and postinfection with *V. parahaemolyticus* bacteria, the antioxidant activity of whiteleg shrimp supplemented with selenoprotein was significantly better (*P* < 0.05) than that of the control group when analyzed by two-way ANOVA. The SOD and GPx enzyme values of whiteleg shrimp supplemented with selenoprotein were significantly higher (*P* < 0.05) than the control as the selenoprotein dose increased in the feed. The MDA value of whiteleg shrimp fed with selenoprotein supplementation showed significantly better *P* < 0.05 than the control. At 48 hours postchallenge test, the SOD and GPx values of whiteleg shrimp decreased in all treatments, while the MDA value increased. The SOD and GPx values of whiteleg shrimp fed with selenoprotein supplementation postchallenge test were significantly (*P* < 0.05) higher than the PC as the selenoprotein dose in the diet increased. The MDA value of whiteleg shrimp fed with selenoprotein supplementation postchallenge test was significantly better (*P* < 0.05) than the PC.

## 4. Discussion

The weight gain of shrimp supplemented with selenoprotein at a dose of 7.5 g kg of feed^−1^, equivalent to the consumption of 2.72 mg Se kg of feed^−1^, was 12.04% greater than control. This result indicates that the greater the dose of selenoprotein added, the higher the weight gain of shrimp, as long as the selenium dose remains within the shrimps' tolerance limit. These findings are consistent with the findings of [36], which demonstrated that organic selenium supplementation in the form of hydroxyl methionine selenium (HMSe) at a dose of 3.6 mg Se kg feed^−1^ resulted in significantly greater weight gain (*P* < 0.05) in whiteleg shrimp *Litopenaeus vannamei* compared to the control and other dose treatments. Furthermore, the supplementation with inorganic selenium (sodium selenite) doses of 0.45 and 0.81 mg Se kg of feed^−1^ resulted in significantly greater growth (*P* < 0.05) in whiteleg shrimp *Litopenaeus vannamei* than the control and other doses (*P* < 0.05) [37]. Similarly, [38] found that supplementing juvenile salmon-bass *Argyrosomus regius* with organic selenium (Se-yeast) at doses of 2.97 and 3.98 mg Se kg feed^−1^ could result in significantly greater weight gain (*P* < 0.05) compared to the control and other doses. As seen by [37], the optimal dose for using inorganic selenium tends to be less than the optimal dose for using organic selenium.

The selenoprotein-supplemented feed contained more moisture than the control feed (Table 1). Consequently, it is necessary to calculate the feed's dry weight to equalize its moisture content. The selenoprotein supplementation in shrimp feed positively affected FCR, PER, and PR parameters. The higher the PER value [36, 38, 39], the more efficiently feed protein is utilized for fish growth [39]. The higher the PR value, the greater the amount of protein stored or absorbed in the body from the protein consumed [17]. The value of dry FCR in whiteleg shrimp fed with selenoprotein supplementation at a dose of 7.5 g kg of feed^−1^ was significantly better (*P* < 0.05) than in other treatments. The FCR on dry feed bases were used since moisture content of the feed with selenoprotein supplementation was higher compared to the control treatment due to application methods of these selenoprotein compounds. In the case of economic conversion value, the use of selenoprotein increases feed efficiency thus reducing the cost required to grow the shrimp. The lower ECR value in the treatment with supplementation of selenium was due to better biomass growth compared to the control. This result demonstrates how feed supplementation can increase feed efficiency and improve performance production of whiteleg shrimp culture.

Selenium is an effective exogenous antioxidant that eliminates and prevents oxidative stress [40]. In addition, selenium plays a role in the GPx enzyme in reducing cell-damaging lipid peroxidation process or MDA production in the animal body [6, 41–43]. GPx, via the reduced glutathione (GSH) pathway, can reduce hydrogen peroxide and fatty acid hydroperoxides in the cytosol and mitochondria to water and alcohol [18, 44]. Selenium can also increase the number of SOD enzymes in the animal body by reducing the amount of superoxide anion if the amount is excessive due to oxidative stress [7, 8, 36, 45]. The amount of ROS that is not excessive plays a critical role in the signalling process of many cells, whereas an excessive amount can damage important biological molecules of the body, including nucleic acids, proteins, and lipids [9, 46].

Whiteleg shrimp supplemented with selenoprotein at a dose of 7.5 g kg of feed^−1^ resulted in higher GPx and SOD enzyme activity and lower MDA values than the control treatments and other doses of selenoprotein before and after the challenge test. These findings are consistent with the findings of Wang et al. [36], which revealed that organic selenium supplementation in the form of hydroxyl methionine selenium (HMSe) could increase the GPx and SOD enzyme activity values and decrease the MDA values of whiteleg shrimp *Litopenaeus vannamei* at doses between 3.52 and 3.7 mg Se kg of feed^−1^. In addition, Wang et al. [47] illustrated that the values of GPx and SOD activity decreased, while MDA increased in serum and the hepatopancreas of juvenile Chinese mitten crab *Eriocheir sinensis* exposed to 2 mg L^−1^ nitrite stress. However, GPx, SOD, and MDA values in serum and the hepatopancreas were better at a dose of 0.5–1 mg Se kg of feed^−1^ organic selenium (Se-yeast).

The study of Ilham et al. [48] described that organic selenium supplementation in the form of selenoamino acids (Se-AA) at a dose of 2 g kg of feed^−1^ could increase the digestibility parameter value of juvenile barramundi *Lates calcarifer* Bloch 1970. It was explained that AA-chelated Se was suspected to be absorbed into the mucosal tissue. Then, along with other essential elements, they are used as cofactors in the synthesis of hydrolytic enzymes, such as gastrointestinal (GI) GPx. The GI-GPx enzyme is the most influential selenoprotein antioxidant enzyme in maintaining the integrity of the intestinal mucosa [49]. In this selenoprotein study, this mechanism is believed to occur in whiteleg shrimp. Selenoprotein supplementation at 7.5 g kg of feed^−1^ resulted in greater ADC values of feed (material), protein, and energy than the control treatment and other doses of selenoprotein. Selenium supplementation was also able to significantly improve animal protein digestibility by increasing the number and activity of protein-degrading microbes in the intestines and proteolytic digestive enzymes [50, 51]. Therefore, supplementation of selenoprotein in feed enables shrimp to digest feed effectively.

Antioxidant activity is positively correlated with the immune response; increased antioxidant activity in selenium-supplemented shrimp will enhance the cellular and humoral immune system [52, 53]). Whiteleg shrimp lacks the immune system commonly found in vertebrate animals (specific immune system), relying instead on nonspecific immune system mechanisms in response to oxidative or environmental stress [54]. It necessitates the administration of immunostimulants to whiteleg shrimp so that their immune system is always prepared to combat pathogens that have the potential to cause disease ([55, 56]. The values of cellular (THC and AF) and humoral (PO, proPO, RB, AL, and PE) immune responses of shrimp supplemented with selenoprotein were greater than those of the controls, indicating better immunity against *V. parahaemolyticus* bacteria. It made the survival of selenoprotein-treated shrimp superior to that of the control group before and after the challenge test.

Hemocytes play an important role in the immune system of crustaceans, which is required for cytotoxicity, cell recognition, melanization, and phagocytosis [57–59]. The total number of hemocytes is a stable immune parameter used to evaluate the stress response, but it is susceptible to changes in intrinsic and extrinsic stimulation [60]. Sritunyalucksana et al. [61] demonstrated that organic selenium supplementation at a dose of 0.88 mg Se kg of feed^−1^ could increase the THC value and survival of Chinese white shrimp *Penaeus chinensis* infected with Taura syndrome virus (TSV). Then, Wang et al. [47] reported that THC and AL of juvenile Chinese mitten crab *Eriocheir sinensis* decreased under nitrite stress at 2 mg L^−1^ but increased with increasing selenium doses (0.5–1 mg Se kg feed^−1^). Lysozyme is one of the key defense components against pathogens and oxidative stress due to the lack of an adaptive immune system in crustaceans [62] and fish [7, 8]. Lysozyme activity is a crucial indicator of innate immunity because it can act as antibacterial, antiviral, and anti-inflammatory [63]. Similar results occurred in the study of Kong et al. [6], which showed that the supplementation of organic selenium (Se-yeast) at doses of 0.31 and 0.47 mg Se kg of feed^−1^ could increase the PO and AL values in giant freshwater prawn *Macrobrachium nipponense*.

The PO enzyme is a sensitivity indicator that indicates the immune status of crustaceans and is crucial for the humoral defense of antibacterial immunity [64]. The study of Chiu et al. [52] showed that supplementation with inorganic selenium (sodium selenate) at doses of 1.54 and 2.19 mg Se kg feed^−1^ and organic selenium (seleno-L-methionine) at doses of 1.58 and 2.21 mg Se kg feed^−1^ could increase the value of PO, RB, and PA and disease resistance against pathogenic bacteria *Debaryomyces hansenii* in giant freshwater prawn *Macrobrachium rosenbergii*. It was stated that the higher PO activity value of selenium-treated shrimp compared to the control indicated higher immunity and directly contributed to the fight against pathogenic bacteria, *D*. *hansenii*, via the melanization process [52]. Supplementation of organic selenium (Se-yeast) at a dose of 2–4 mg Se kg of feed^−1^ can also increase the resistance of yellowtail kingfish to *Vibrio anguillarum* bacteria [65]. Crustaceans possess only nonspecific innate immunity (no other immune defenses), including prophenoloxidase-activating defense system (proPO system), serine protease clotting process, action of endogenous antimicrobial peptides, and phagocytic activity [66, 67].

In response to oxidative stress, crustaceans have increased oxygen radical secretion [56, 68]. Respiratory burst (rapid release of ROS) is beneficial for boosting immunity against pathogenic bacterial infections and boosting the antimicrobial capacity of crustaceans [52, 56, 69]. During phagocytosis, ROS are produced as a defense mechanism against foreign particles, one of which is pathogen infection [70]. Phagocytosis is the process of hemocyte cells engulfing foreign particles in the internal environment, which is essential for eliminating invading pathogens and apoptotic cells [71]. Fuandila et al. [72] reported that the increase in the proportion of PA corresponded with the increase in hemocyte performance, so that the ability to phagocytize foreign particles that entered the body increased. In addition, it was stated that the increase in THC and PA increased the amount of ROS, the main product of RB.

The proPO system is the most important immune system in crustaceans [55, 73]. The active proPO system and numerous other molecules execute a self-defense response, including melanin formation, non-self-recognition, adhesion, and cell-to-cell communication [33, 74–77]. PO is a terminal enzyme of the proPO system; therefore, PO activity can indicate the activation status of the proPO system [52]. PE is produced concurrently with the activation of the proPO system [66]. PE is synthesized in semigranular or granular hemocytes, stored in the granules of the secretory cells and then released during the degranulation process [78]. Active PE mediates the adhesion of semigranular and granular hemocytes [79], peroxidase activity [80], improvement of encapsulation [81], and stimulation of phagocytosis [82].

Johansson et al. [83] described partial amino acid sequencing and cDNA cloning of peroxinectin-protein homologs with CuZn-containing superoxide dismutase (CuZnSOD). Furthermore, enzyme staining revealed that the protein possessed SOD activity and concluded that the PE binding protein was extracellular SOD (EC-SOD), so that the binding of cell surface SOD to cell adhesion PE can mediate or regulate cell adhesion and phagocytosis processes, and is essential for production of reliable local defense substances. This is consistent with this selenoprotein supplementation study; the values of SOD and peroxinectin increased with increasing selenoprotein dose in the feed.

In conclusion, from this research it can be seen that supplementation of selenoprotein at 7.5 g kg feed^−1^ improves the digestibility, ADG, SGR, FCR, PER, PR, and antioxidant capacity of whiteleg shrimp. Furthermore, supplementation of selenoprotein at 7.5 g kg feed^−1^ also improved survival upon challenge with *V. parahaemolyticus* and demonstrated higher THC, PA, LA, RB, and PO value compared to the control treatment.

## Figures and Tables

**Table 1:** Results of proximate analysis and selenium analysis of test feed.

Composition	Doses of selenoprotein (g kg feed^−1^) in wet weight (dry weight)			
	0	2.5	5	7.5
Moisture (%)	8.66	19.38	19.23	19.38
Protein (%)	31.97 (35.00)	28.29 (35.10)	28.43 (35.20)	28.45 (35.29)
Lipid (%)	5.53 (6.06)	4.95 (6.14)	4.99 (6.18)	5.02 (6.23)
Ash (%)	9.10 (9.96)	8.13 (10.08)	8.18 (10.12)	8.19 (10.16)
Crude fiber (%)	3.58 (3.92)	2.94 (3.64)	2.97 (3.68)	2.99 (3.71)
NFE (%)^1^	41.16 (45.07)	36.30 (45.03)	36.20 (44.82)	35.98 (44.62)
Se (mcg se g^−1^)	0.77	1.27	2.08	2.72
GE (kcal kg^−1^)^2^	4397.85	4410.04	4410.77	4411.77
C/P (kcal g^−1^)^3^	12.57	12.56	12.53	12.50

^1^Wet nitrogen-free extract (NFE) = 100 − (moisture + protein + lipid + ash + crude fiber) and dry NFE = 100 − (protein + lipid + ash + crude fiber). ^2^Gross energy (GE) composition of dry feed was calculated based on protein = 5.64 kcal g protein^−1^, lipid = 9.44 kcal g lipid^−1^, and carbohydrates or NFE = 4.11 kcal g carbohydrate^−1^ [26]. ^3^C/P = calories/protein.

**Table 2:** Digestibility performance of whiteleg shrimp after being given treatment feed.

Observation parameters^1^	Doses of selenoprotein (g kg feed^−1^)			
	0	2.5	5	7.5
ADC of material (%)	51.97 ± 1.33^a^	52.74 ± 1.80^ab^	54.87 ± 0.41^b^	54.87 ± 0.91^b^
ADC of protein (%)	80.95 ± 1.29^a^	81.94 ± 1.45^ab^	82.61 ± 1.53^ab^	83.88 ± 1.48^b^
ADC of energy (%)	65.91 ± 1.06^a^	67.71 ± 1.17^b^	68.66 ± 0.44^b^	68.69 ± 0.62^b^

^1^Apparent digestible coefficient (ADC). ^2^Mean ± standard deviation. ^3^Different letters in the same row indicate significantly different treatment effects (*P* < 0.05). The values shown are the mean and standard deviation.

**Table 3:** Growth performance of whiteleg shrimp after being fed treatment for 70 days.

Observation parameters^1^	Doses of selenoprotein (g kg feed^−1^)			
	0	2.5	5	7.5
*B*_0_ (g)	22.95 ± 0.10	22.95 ± 0.06	22.98 ± 0.10	22.98 ± 0.10
*B*_*t*_ (g)	109.53 ± 0.60^a^	113.25 ± 0.57^a^	125.67 ± 3.38^b^	135.37 ± 2.91^c^
*W*_0_ (g)	1.53 ± 0.01	1.53 ± 0.01	1.53 ± 0.01	1.53 ± 0.01
*W*_*t*_ (g)	9.13 ± 0.05^a^	9.44 ± 0.05^a^	9.67 ± 0.26^ab^	10.17 ± 0.50^b^
SR (%)	81.67 ± 3.33	81.67 ± 3.33	83.33 ± 6.67	86.67 ± 5.44
FI (g)	178.62 ± 2.33^a^	196.15 ± 6.09^b^	202.27 ± 2.57^bc^	206.58 ± 7.20^c^
Dry FI (g)	163.15 ± 2.13^ab^	158.13 ± 4.91^a^	163.37 ± 2.08^ab^	166.55 ± 5.80^b^
FCR	1.82 ± 0.05^b^	1.87 ± 0.03^b^	1.82 ± 0.05^b^	1.72 ± 0.03^a^
Dry FCR	1.66 ± 0.04^c^	1.51 ± 0.03^b^	1.47 ± 0.04^b^	1.39 ± 0.03^a^
SGR (%)	2.58 ± 0.01^a^	2.63 ± 0.01^a^	2.66 ± 0.04^ab^	2.74 ± 0.08^b^
ADG (g/day)	0.109 ± 0.01^a^	0.113 ± 0.01^a^	0.116 ± 0.01^ab^	0.123 ± 0.01^b^
PER	1.52 ± 0.03^a^	1.63 ± 0.06^b^	1.79 ± 0.04^c^	1.91 ± 0.08^d^
PR (%)	23.93 ± 0.46^a^	26.01 ± 0.93^b^	28.98 ± 0.71^c^	31.20 ± 1.26^d^

^1^Initial biomass (*B*_0_), final biomass (*B*_*t*_), individual initial weight (*W*_0_), final individual weight (*W*_*t*_), survival rate (SR), feed intake (FI), feed conversion ratio (FCR), specific growth rate (SGR), average daily growth (g/day), protein efficiency ratio (PER), and protein retention (PR). ^2^Mean ± standard deviation. ^3^Different letters in the same row indicate significantly different treatment effects (*P* < 0.05). The values shown are the mean and standard deviation.

**Table 4:** Economic conversion ratio (ECR) of each selenoprotein treatment.

Doses of selenoprotein (g kg feed^−1^)	Price of supplementation per kg feed	Price of treatment feed per kg	Dry FCR	ECR ($ per kg)^1^
0	—	1.05$	1.66	1.74$
2.5	0.020$	1.07$	1.51	1.62$
5	0.041$	1.09$	1.47	1.60$
7.5	0.061$	1.11$	1.39	1.54$

^1^Economic conversion ratio (ECR) = price of treatment feed per kg × dry FCR. ^2^Price of AQUACELL SF = Rp.125.000 kg^−1^; price of commercial feed = Rp.16.000 kg^−1^; currency conversion 1 USD = Rp 15.253,94.

**Table 5:** Immune response, immune gene expression, and antioxidant activity of whiteleg shrimp after feeding treatment and infection with *V*. *parahaemolyticus* and resistance of shrimp after infection.

Observation parameters^1^	Sampling time (hour)^4^	Doses of selenoprotein (g kg feed^−1^)				
		0 (-)^3^	0 (+)^3^	2.5	5	7.5
Immune response						
SR postinfection (%)		100 ± 0.00^c^	45 ± 7.07^a^	75 ± 7.07^b^	80 ± 14.14^b^	80 ± 0.00^b^
THC (cells mL^−1^) × 10^6^	0	6.35 ± 0.07^a^	—	15.30 ± 0.01^b^	17.20 ± 0.03^c^	19.80 ± 0.06^d^
	48	6.20 ± 0.42^b^	3.25 ± 0.35^a^	10.10 ± 0.01^c^	11.60 ± 0.06^d^	13.40 ± 0.07^e^
PO (100 *μ*L^−1^)	0	0.09 ± 0.07^a^	—	0.12 ± 0.08^b^	0.13 ± 0.09^bc^	0.15 ± 0.01^c^
	48	0.09 ± 0.06^d^	0.06 ± 0.06^a^	0.07 ± 0.01^ab^	0.08 ± 0.01^bc^	0.08 ± 0.04^cd^
RB (10 *μ*L^−1^)	0	0.15 ± 0.02^a^	—	0.33 ± 0.01^b^	0.47 ± 0.01^c^	0.58 ± 0.01^d^
	48	0.14 ± 0.01^bc^	0.10 ± 0.03^a^	0.12 ± 0.01^b^	0.15 ± 0.01^c^	0.17 ± 0.01^d^
PA (%)	0	39 ± 0.04^a^	—	61 ± 0.06^b^	68 ± 0.04^b^	67 ± 0.02^b^
	48	38 ± 0.03^ab^	30 ± 0.01^a^	40 ± 0.04^b^	49 ± 0.02^c^	50 ± 0.04^c^
LA (unit/mL)	0	31.84 ± 1.18^a^	—	51.67 ± 0.94^b^	83.5 ± 1.17^c^	102.5 ± 1.66^d^
	48	29.00 ± 1.41^b^	16.67 ± 0.94^a^	36.00 ± 0.95^c^	46.84 ± 1.18^d^	68.00 ± 1.89^e^
Relative expression of mRNA immune gene						
proPO	48	0.44 ± 0.01^a^	0.49 ± 0.01^a^	0.88 ± 0.07^b^	0.92 ± 0.06^b^	1.01 ± 0.05^c^
PE	48	0.43 ± 0.01^a^	0.44 ± 0.01^a^	0.92 ± 0.01^b^	0.96 ± 0.04^b^	1.03 ± 0.02^c^
Antioxidant activity						
SOD (U/mg protein)	0	7.06 ± 0.04^a^	—	9.21 ± 0.09^b^	10.26 ± 0.33^c^	11.19 ± 0.04^d^
	48	7.29 ± 0.15^b^	6.72 ± 0.21^a^	8.59 ± 0.13^c^	9.28 ± 0.05^d^	9.90 ± 0.09^e^
GPx (U/mg protein)	0	59.24 ± 0.11^a^	—	72.96 ± 0.06^b^	79.19 ± 2.53^c^	83.54 ± 0.92^d^
	48	58.63 ± 0.86^b^	50.82 ± 0.14^a^	60.13 ± 1.01^b^	66.69 ± 0.81^c^	71.16 ± 1.33^d^
MDA (nmol/mg protein)	0	0.11 ± 0.01^b^	—	0.08 ± 0.02^a^	0.08 ± 0.02^a^	0.08 ± 0.01^a^
	48	0.11 ± 0.01^a^	0.13 ± 0.02^b^	0.11 ± 0.01^a^	0.11 ± 0.03^a^	0.11 ± 0.02^a^

^1^Survival rate (SR), total hemocyte count (THC), phenoloxidase (PO), respiratory burst (RB), phagocytic activity (PA), lysozyme activity (LA), prophenoloxidase (proPO), peroxinectin (PE), glutathione peroxidase (GPx), superoxide dismutase (SOD), and malondialdehyde (MDA). ^2^Mean ± standard deviation. ^3^Different letters in the same row indicate significantly different treatment effects (*P* < 0.05). The values shown are the mean and standard deviation. ^4^(-)/NC: no bacterial infection *V. parahaemolyticus* and; (+)/PC: bacterial infection *V. parahaemolyticus*; NC: negative control; PC: positive control. ^5^0 hours: prior challenge test and 48 hours: 48 hours postchallenge test.

## Data Availability

The data that support the findings of this study are available from the corresponding author upon reasonable request.
